# Duodenoscopy-assisted diagnosis and management of a duodenal varix

**DOI:** 10.1016/j.vgie.2024.11.007

**Published:** 2024-11-29

**Authors:** Kar Wai Lau

**Affiliations:** Royal Stoke University Hospital, University Hospitals of North Midlands NHS Trust, United Kingdom

## Background

Duodenal varices account for 1% to 3% of all varices in patients with cirrhosis.^1^^,^^2^ It is a type of ectopic varix that occurs when collateral veins develop between the portal vein trunk or superior mesenteric vein and the inferior vena cava. The most common site of involvement is the duodenal bulb followed by the second part of the duodenum.^3^^,^^4^ Bleeding from duodenal varices is rare but is generally massive and has a reported mortality rate of up to 40%.^5^ Additionally, the condition is also difficult to diagnose and control, with endoscopic treatment frequently used as first-line treatment.^5^ Alternative radiologic treatment options for duodenal varices include transjugular intrahepatic portosystemic shunt (TIPSS) placement and balloon-occluded retrograde transvenous obliteration. Here, we describe a case of severe upper GI hemorrhage due to a bleeding duodenal varix that was identified and treated at the index endoscopic procedure.

## Case presentation

A 67-year-old woman with no significant medical history presented with hematemesis and peri-arrest state. She had a history of excess alcohol intake (40 units per week) and there was a suspicion of variceal bleeding. Her laboratory parameters were as follows: hemoglobin 65 g/L, platelet 61 × 10^9^/L, INR 2.0, total bilirubin 49 μmol/L, and albumin 28 g/L.

**Figure 1:**
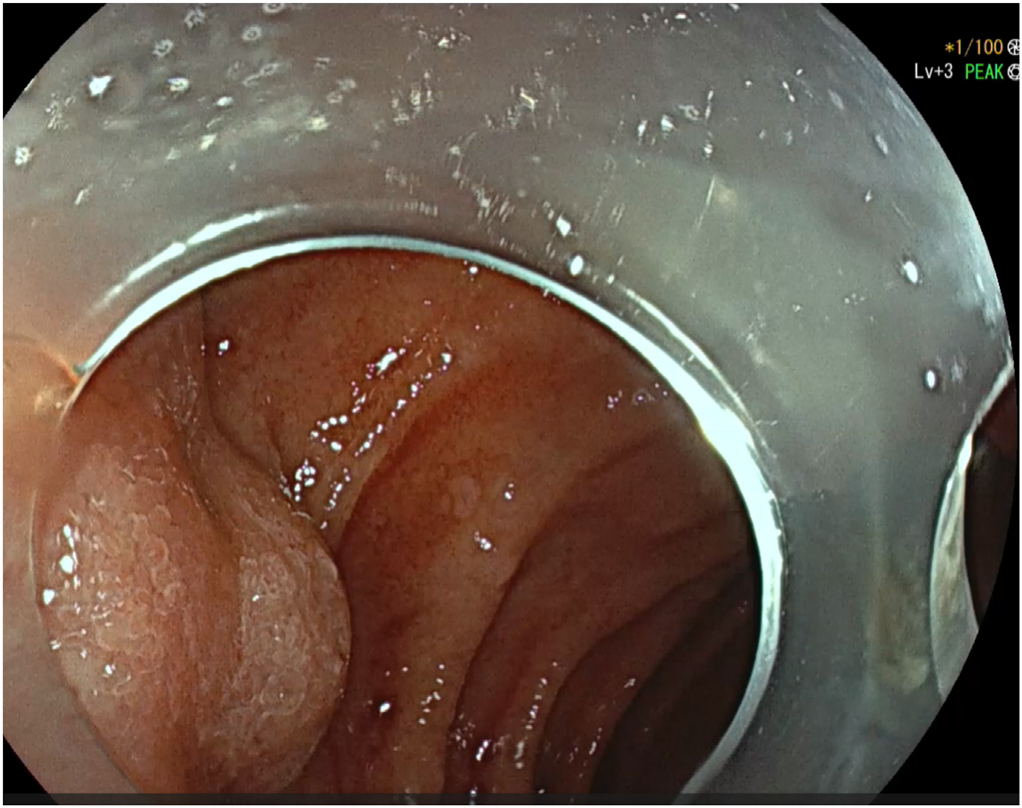
View of submucosal abnormality at the D1-D2 junction with a gastroscope.

**Figure 2:**
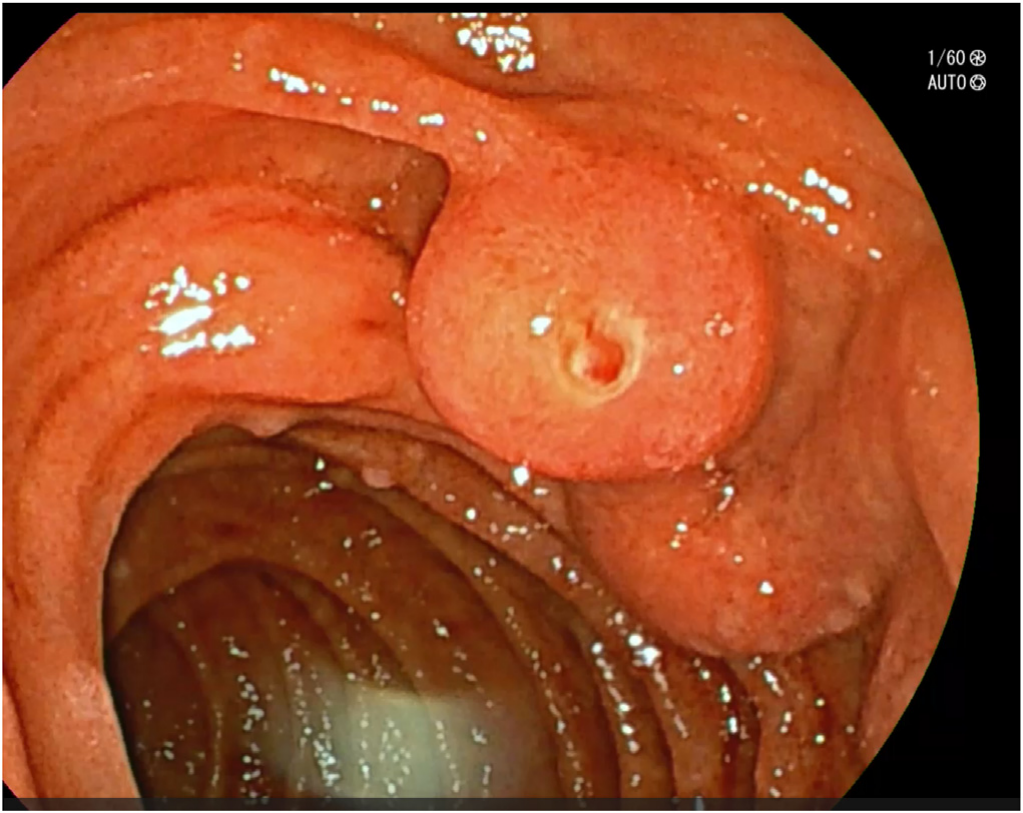
Clear view of the duodenal varix with a side-viewing duodenoscope.

**Figure 3:**
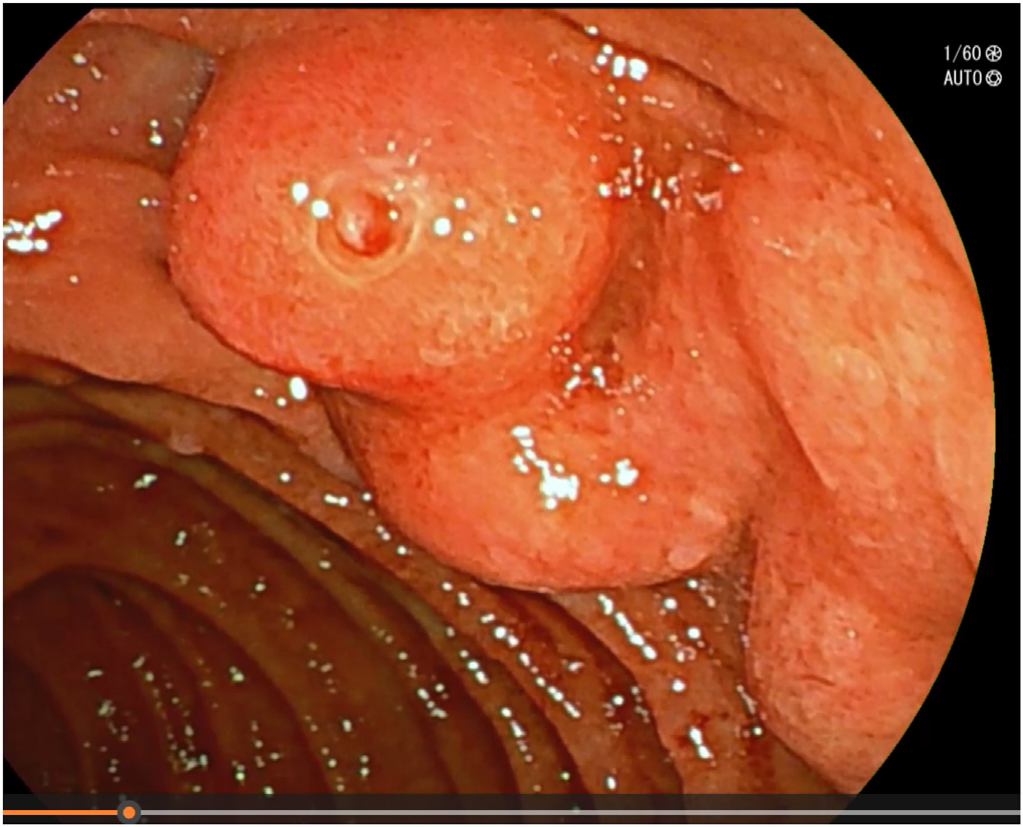
Another view of the duodenal varix with a side-viewing duodenoscope.

After adequate resuscitation with intravenous fluids and blood products, an EGD was performed with the patient under general anesthesia with a gastroscope, but a source of bleeding was not positively identified in the esophagus or stomach. Fresh blood was seen in the duodenal bulb and descending duodenum and there was an impression of a submucosal abnormality at the D1-D2 junction (Fig. 1). Second endoscopy with a duodenoscope revealed a large duodenal varix with stigmata of recent hemorrhage (Figs. 2 and 3). As the patient was hemodynamically unstable, we proceeded to treat the varix with *n*-butyl-2-cyanoacrylate (Histoacryl, B. Braun, Melsungen, Germany) prepared in the following manner (Video 1, available online at www.videogie.org).

a) Histoacryl 0.5 mL diluted with ethiodized oil (Lipiodol, Guerbet, Villepinte, France) 0.5 mL in a 1:1 ratio drawn in a 3-mL syringe. The dilution of Histoacryl in this manner modulates the rate of glue polymerization and achieves the best outcome in our experience.^6^
b) Distilled water (2 mL) is drawn in a 3-mL syringe.
c) Both syringes are connected to a 3-way stopcock and injection needle. This allows rapid sequential injection of Histoacryl-Lipiodol and distilled water.
d) A 23-gauge injection needle is primed with distilled water.
e) The endoscope working channel is flushed with Lipiodol before insertion of the injection needle to avoid glue damage.
f) The varix is punctured with the needle, followed by injection of the glue-lipiodol mixture.
g) The endoscope is immediately flushed with 2 mL distilled water by turning the stopcock valve.
h) The above steps are repeated until the varix is obliterated.
i) Once the therapy is completed, the endoscope is removed with the tip of the needle sheath extending outside the working channel. The tip is cut outside the working channel to prevent residual glue from being introduced into the working channel of the endoscope.

**Figure 4:**
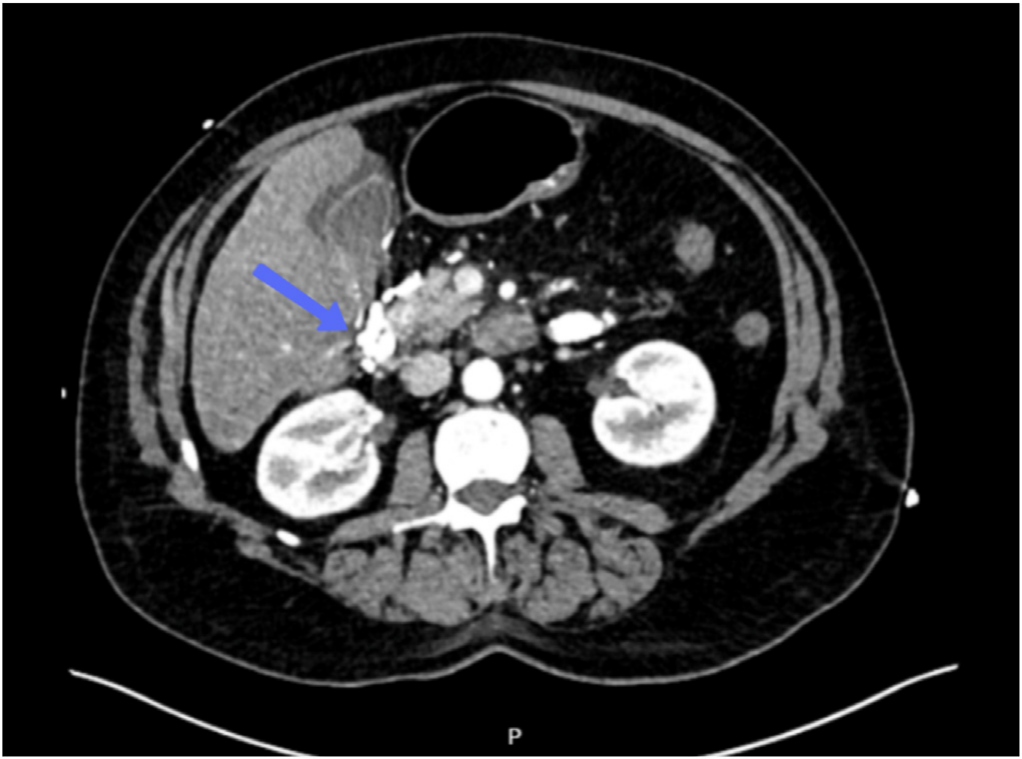
Postendotherapy computed tomographic scan showing Histoacryl-Lipiodol mixture at the site of the duodenal varix.

**Figure 5:**
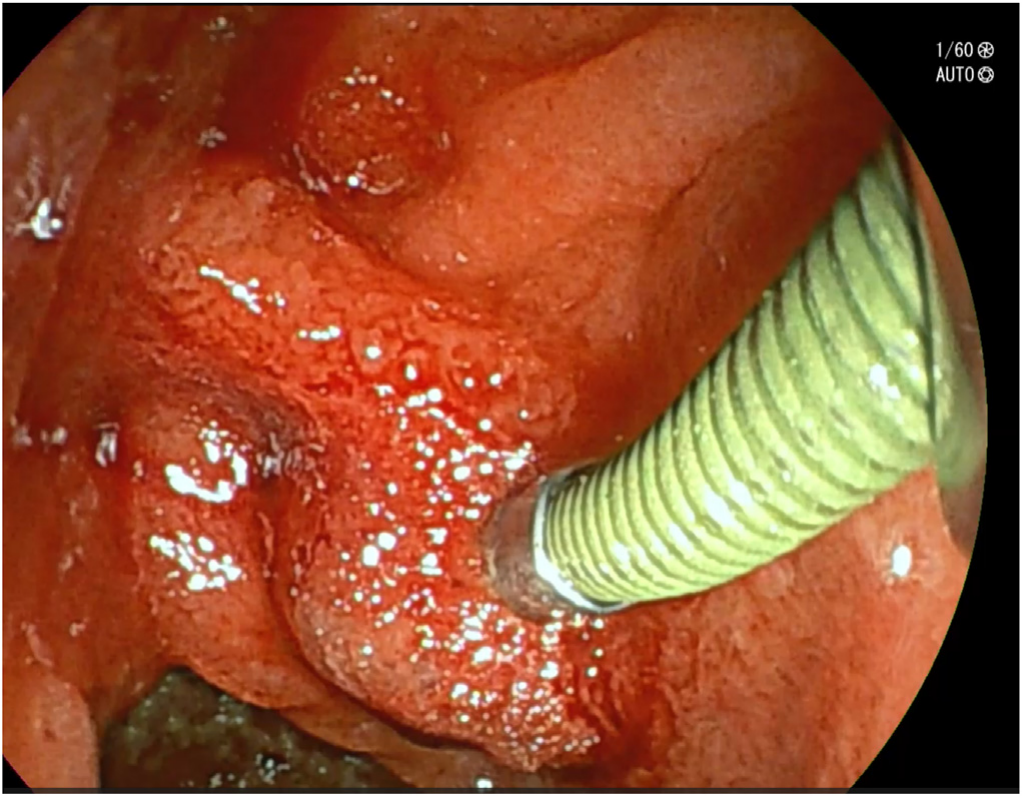
Duodenal varix after injection with Histoacryl-Lipiodol.

Subsequent triple-phase abdominal computed tomography (Fig. 4, Video 1, available online at www.videogie.org) and endoscopic ultrasonography demonstrated complete obliteration of the duodenal varix. Post injection, there was noticeable visual hardening and solidification of the varix, with additional firmness noted on palpation with the needle sheath (Fig. 5). She was additionally treated with intravenous terlipressin and antibiotics with no further bleeding noted and hemodynamic stability. The patient was subsequently transferred to a neighboring facility for the evaluation of TIPSS placement.

A transjugular liver biopsy with hepatic venous pressure gradient was performed, which confirmed the presence of clinically significant portal hypertension (10 mm Hg) despite treatment with carvedilol 6.25 mg daily. Because of successful endoscopic treatment, subsequent TIPPS was considered unnecessary. She remained well and was discharged 11 days after her initial presentation. She had no further bleeding episodes in 5 months of follow-up.

In patients presenting with upper GI hemorrhage, it is worthwhile using a duodenoscope for complementary assessment if the source of bleeding is obscure using a gastroscope. Use of a duodenoscope also provided an optimal therapeutic opportunity with an en-face view and stable approach to the target pathology. Finally, endoscopic treatment for bleeding duodenal varices is safe and effective, especially in the setting of life-threatening clinical presentation.

## Disclosures

None of the authors have any disclosures to make.

## Supplementary data

Video 1 XXX.
